# LDK378 improves micro- and macro-circulation via alleviating STING-mediated inflammatory injury in a Sepsis rat model induced by Cecal ligation and puncture

**DOI:** 10.1186/s12950-019-0208-0

**Published:** 2019-02-18

**Authors:** Weiwei Ge, Qiaohua Hu, Xiangshao Fang, Juanhua Liu, Jing Xu, Juntao Hu, Xuefen Liu, Qin Ling, Yue Wang, He Li, Ming Gao, Longyuan Jiang, Zhengfei Yang, Wanchun Tang

**Affiliations:** 1 The Second Affiliated Hospital of Anhui Medical University, Hefei, China; 2 Sun Yat-sen Memorial Hospital, Sun Yat-sen University, 107 Yan Jiang Xi Road, Guangzhou, 510120 China; 3 The eastern hospital of the first affiliated hospital, Sun Yat-sen University, Guangzhou, China; 4 The First Affiliated Hospital of Xi’an Jiaotong University, Xi’an, China; 5 The first Affiliated Hospital of Guangxi Medical University, Nanning, China; 6 Weil Institute of Emergency and Critical Care Research, School of Medicine, Virginia Commonwealth University, Richmond, VA USA

**Keywords:** LDK378, Sepsis, ALK-STING pathway, Inflammation

## Abstract

**Background:**

Sepsis is a systemic inflammatory response syndrome caused by severe infections. LDK378, a second-generation ALK inhibitor, exhibits a potential anti-inflammatory function against sepsis. Micro- and macro-circulatory dysfunctions are pivotal elements of the pathogenesis of severe sepsis and septic shock. We hypothesized that LDK378 can improve micro- and macro-circulation of septic rats, therefore improving the outcome of survival via blocking the ALK-STING pathway to attenuate inflammatory injuries.

**Methods:**

A septic rat model was established by the cecal ligation and puncture (CLP) method. A total of 60 rats were randomized into three groups: a sham group, CLP group, and CLP + LDK378 group (*n* = 20 in each group). Five rats were randomly selected from each group for the mechanism study; the remaining 15 rats in each group were involved in a survival curve examination. A sidestream dark field video microscope was used to record sublingual microcirculation and mean arterial pressure (MAP) and levels of inflammatory cytokine secretion were examined at 6 h, 30 h, and 54 h after CLP surgery. Expressions of TANK binding kinase 1 (TBK1) and its downstream targets were determined, and histological alterations to the heart, lungs, and kidneys were examined at 54 h after CLP surgery.

**Results:**

We found the group that received LDK378 treatment showed increased MAP levels compared to the CLP group at 30 h and 54 h. Meanwhile, LDK378 ameliorated the perfused small vessel density and microvascular flow index, decreased the expression of TNF-a and IL-6, and upregulated the expression of IL-10 in comparison with the CLP group. LDK378 injections also downregulated the expression of TBK1 and its downstream targets. Furthermore, LDK378 treatment significantly reduced sepsis-induced organ injuries, therefore improving survival rates.

**Conclusions:**

These findings demonstrate that LDK378 treatment can improve microcirculation and reduce organ injuries in CLP-induced septic rats via the regulation of inflammatory cytokine secretion and the downstream signaling components of the ALK-STING pathway.

## Background

Sepsis is defined as a life-threatening organ dysfunction caused by a dysregulated host response to infection [1, 2]. It is an acute systemic reaction to microbes that invade the body, accompanied by a strong innate immune response [3]. With the stimulation of pathogens, proinflammatory cytokines are released, generating systemic inflammatory responses that impair the micro- and macro-circulation function [4, 5]. Septic shock is a subset of sepsis with hemodynamic alterations and metabolic dysfunction associated with organ dysfunction. Alterations in microcirculatory blood flow have been identified in severe sepsis [6] and the severity of theses alteration is associated with a poor outcome [7].

Sepsis remains the primary cause of death in critical care units despite the use of modern antibiotics, ventilator management, and resuscitative therapies, affecting millions of people all over the world each year. There are currently no existing FDA-approved treatment options for sepsis, though many therapeutic clinical trials have been conducted. Mortality rates from sepsis are 41% in Europe and 28.3% in the United States [8]. The cost of sepsis has become the most expensive health-care problem in the United States, at more than 20 billion dollars annually. [9]

New insights into immune dysregulation illustrate an enduring inflammatory state driven by a dysfunctional innate and suppressed adaptive immunity that culminates in persistent organ injury and death. This initial inflammatory process, if not abated, also contributes to organ failure and early mortality [10, 11]. Inflammatory and anti-inflammatory responses, as well as innate and adaptive immune systems, represent potential targets for immune therapy to improve sepsis outcomes. Inflammatory response is mediated by the production of proinflammatory cytokines such as tumor necrosis factor TNF = a, IL-1 and IL-6. However, this early response is followed by a transition to sustained production of anti-inflammatory mediators such as IL-10. Animal studies of sepsis suggested a largely unopposed proinflammatory response to severe sepsis resulted in increased organ injury and mortality, whereas a greater anti-inflammatory response resulted in less severe sepsis.

Stimulator of interferon genes (STING) is an endoplasmic reticulum (ER)-resident membrane protein which mediates cytosolic pathogen DNA-induced innate immunity and inflammatory responses in host defenses [12]. STING is activated by cyclic dinucleotide, then translocates to the Golgi apparatus, an event that triggers STING assembly with the downstream enzyme TANK-binding kinase 1 (TBK1). This assembly leads to the phosphorylation of the transcription factor interferon regulatory factor 3 (IRF3), Phosphorylated IRF3 forms a homo-dimer to enter the nucleus and functions together with NF-kB (p65) to induce type-I interferon (IFN) and other pro-inflammatory cytokines [13]. STING was identified by investigators screening cDNA libraries for genes that, when overexpressed, were sufficient to activate the production of IFN [14]. Further studies have revealed STING-knockout mice are susceptible to lethal infection, demonstrating the critical role of STING in facilitating an immune response to pathogens [15].

The 3′3’-cGAMP is a type of cyclic dinucleotide (CDNs) and serve as a canonical STING ligand to induce the production of type I IFNs. LDK378, a novel ALK inhibitor approved for advanced-stage non-small cell lung cancer (NSCLC) with ALK gene rearrangement, was recently found to have the effect of blocks 3′3’-cGAMP-induced IFNβrelease in immortalized bone marrow-derived macrophages (iBMDMs). Similar to pharmacological ALK inhibition, genetic inhibition of ALK also attenuated STING ligand-induced expression and release in IBMDMs. ALK seem to play an important role in the regulation of STING signaling pathway [16].

LDK378, a novel ALK inhibitor approved for advanced-stage non-small cell lung cancer (NSCLC) with ALK gene rearrangement, exhibited promising anti-inflammatory activity in animal models of lethal sepsis. Zeng, L. et al. [16] investigated macrophage activation in an in-vitro study and found that ALK inhibition increased septic animal survival in STING-deficient mice in vivo. Activation of the STING pathway by ALK may be a crucial step for its immunological activity and may contribute to the pathogenesis of sepsis.

The cecal ligation and puncture (CLP) model [17, 18] is considered the gold standard in sepsis research. In a CLP model, sepsis develops due to peritoneal contamination with mixed flora in the presence of devitalized or ischemic tissue, thus the resemblance to clinical reality. Furthermore, metabolic, immunological, and apoptotic responses are similar in the CLP model and in human disease.

Microcirculatory dysfunction is a pivotal element of the pathogenesis of sepsis. Because the microcirculation is the primary site of oxygen and nutrient exchange, therapeutic interventions aimed at increasing organ perfusion should be accompanied by improved microvascular perfusion.

This study aimed to demonstrate two hypotheses: first, LDK378 can alleviate microcirculation and hemodynamics of CLP-induced septic rats, thereby mitigating organic injuries and improving the survival rate. Second, LDK378 blocks the STING pathway activation through interfering with TBK-1-mediated signaling transduction.

## Results

### Mean arterial pressure

Mean arterial pressure in the CLP and LDK378 treatment groups decreased significantly at 6 h after the CLP surgery compared to the sham group. At 30 h and 54 h, mean aortic pressure in the LDK378 treatment group was significantly increased compared to the CLP group (*p* < 0.05) (Table 1).

**Table 1:** Changes of macro- and microcirculatory parameters at baseline, 6 h, 30 h and 54 h. Values are presented as mean ± SD

Group	Sham	CLP	CLP + LDK378
MAP (mmHg)			
BL	124 ± 5.70	124.8 ± 6.91	123.2 ± 4.38
6 h	120 ± 6.78	99.6 ± 9.24^*^	98 ± 4.74^*^
30 h	113.4 ± 11.72	69.6 ± 12.93^*^	86.4 ± 5.68^*#^
54 h	115.2 ± 6.38	46 ± 13.08^*^	74.4 ± 7.06^*#^
PVD (mm/mm^2^)			
BL	18.91 ± 0.756	18.24 ± 0.62	18.63 ± 0.51
6 h	18.9 ± 0.56	16.44 ± 2.61^*^	16.87 ± 1.14
30 h	18.45 ± 0.45	13.56 ± 2.19^*^	16.14 ± 1.33^*#^
54 h	18.63 ± 0.86	12.45 ± 2.41^*^	15.49 ± 1.01^*#^
MFI			
BL	2.9 ± 0.14	2.9 ± 0.14	2.95 ± 0.11
6 h	2.85 ± 0.14	2.55 ± 0.27	2.6 ± 0.45
30 h	2.7 ± 0.11	1.85 ± 0.22^*^	2.3 ± 0.33^*#^
54 h	2.55 ± 0.21	1.5 ± 0.18^*^	2.05 ± 0.33^*#^

*BL* indicates baseline, *MAP* mean arterial pressure, *MFI* microcirculatory flow index, *PVD* perfused vessel density. *p < 0.05 compared with Sham group; #*p* < 0.05 compared with CLP group

### Sublingual microcirculation examination

PVD in the CLP and LDK378 treatment groups significantly decreased in parallel with the reduced MFI compared to the sham group at 6 h. The sublingual microcirculation flow progressively decreased in the CLP group at 30 h and 54 h, however, microcirculation was significantly improved in the LDK378 treatment group (*p* < 0.05) at 30 h and 54 h (Table 1).

### Inflammatory cytokines

**Fig. 1:**
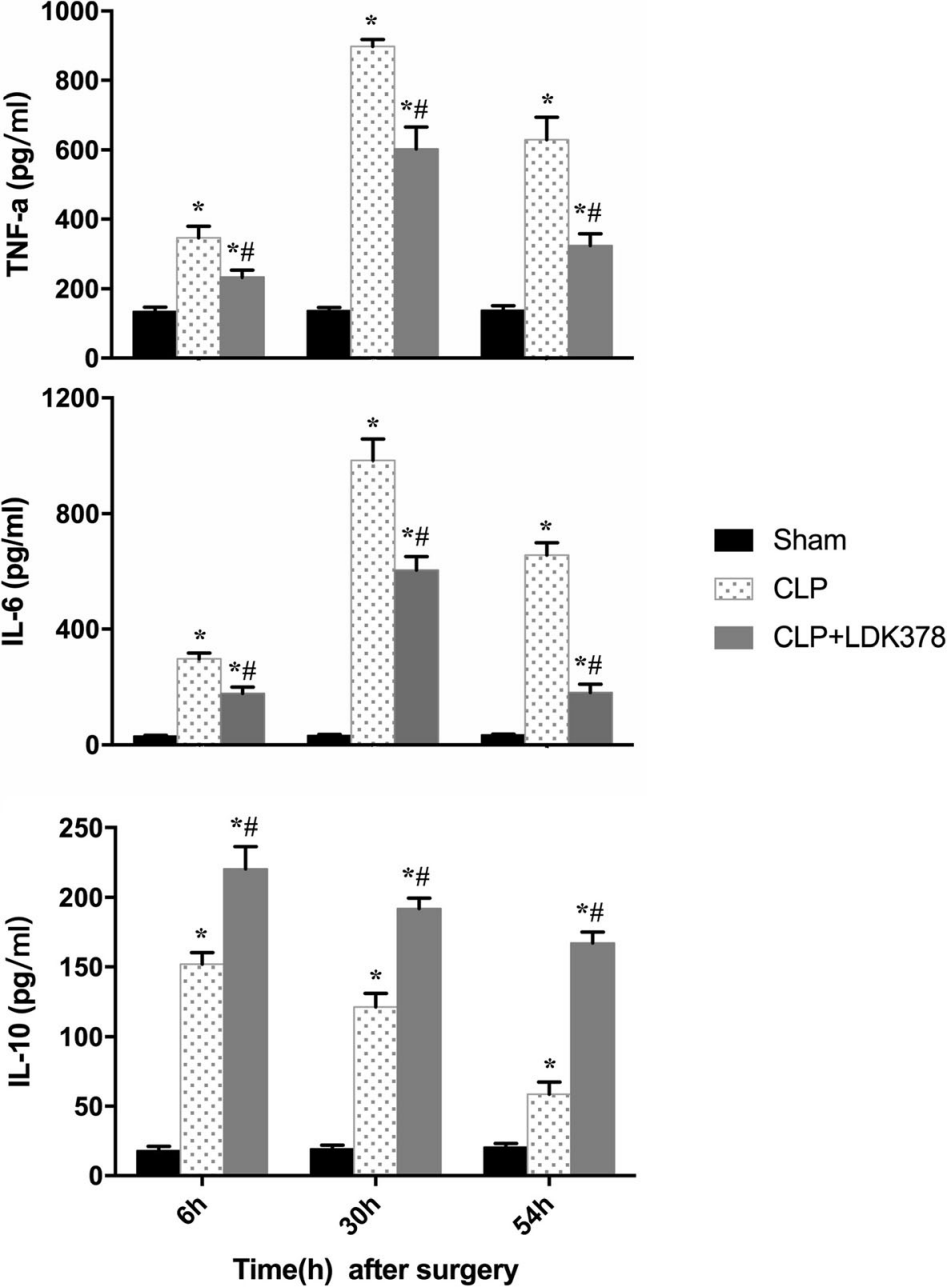
Changes in the biochemical parameters of the serum at different time points; TNF-a, tumor necrosis factor alpha; IL-6, interleukin-6; IL-10, interleukin-10. *p < 0.05 vs sham group; #*p* < 0.05 compared vs CLP group

Sepsis is characterized by the uncontrollable release of pro-inflammatory and anti-inflammatory cytokines. In the present study, two pro-inflammatory cytokines, TNF-a and IL-6 and one anti-inflammatory cytokine, IL-10 were detected at 6, 30, and 54 h after CLP surgery using ELISA kits. Serum levels of TNF-a, IL-6, and IL- 10 at 6 h and 30 h were significantly elevated in CLP-induced sepsis in comparisons with those in the sham group (*p* < 0.05), serum levels of TNF-a, IL-6, and IL- 10 of LDK378 group at 54 h were higher than sham group (*p* < 0.05). The concentrations of TNF-a, IL-6, and IL-10 at 54 h were significantly decreased in the CLP group compared to 30 h (*p* < 0.05). Levels of TNF-a and IL-6 were noticeably higher in the CLP group compared with the LDK378 treatment group. However, the level of IL-10 in the LDK378 treatment group was much higher than in the CLP group (*p* < 0.05) (Fig. 1).

### Western blot analysis

**Fig. 2:**
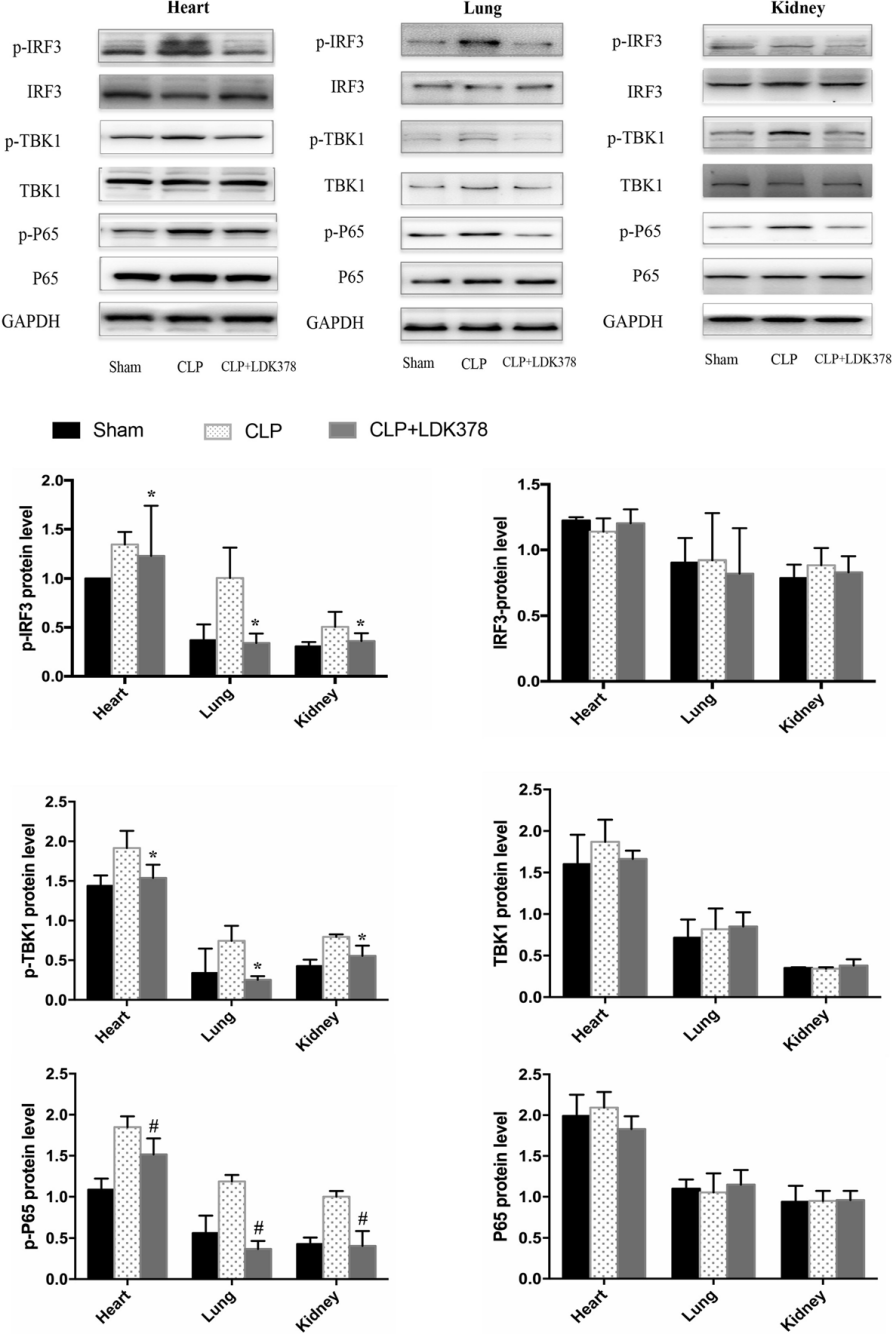
Western blot analysis of indicated protein expression; (**a**) Western blot analysis of indicated protein expression in lung tissues; (**b**) Western blot analysis of indicated protein expression in kidney tissues; *p < 0.05 vs CLP group; #*p* < 0.01 vs CLP group

At 54 h, the levels of p-IRF3 and its downstream targets of p-TBK1 and p-P65 were decreased in heart tissue of the LDK378 treatment group compared to the CLP group. However, levels of total IRF3, TBK1, and P65 did not change in the LDK378 treatment group. Similar to lung and kidney tissues, levels of p-IFR3, p-TBK1 and p-P65 were decreased in the LDK378 treatment group, but levels of total IRF3, TBK1, and P65 did not change in the LDK378 treatment group (Fig. 2).

### Histopathological examination

**Fig. 3:**
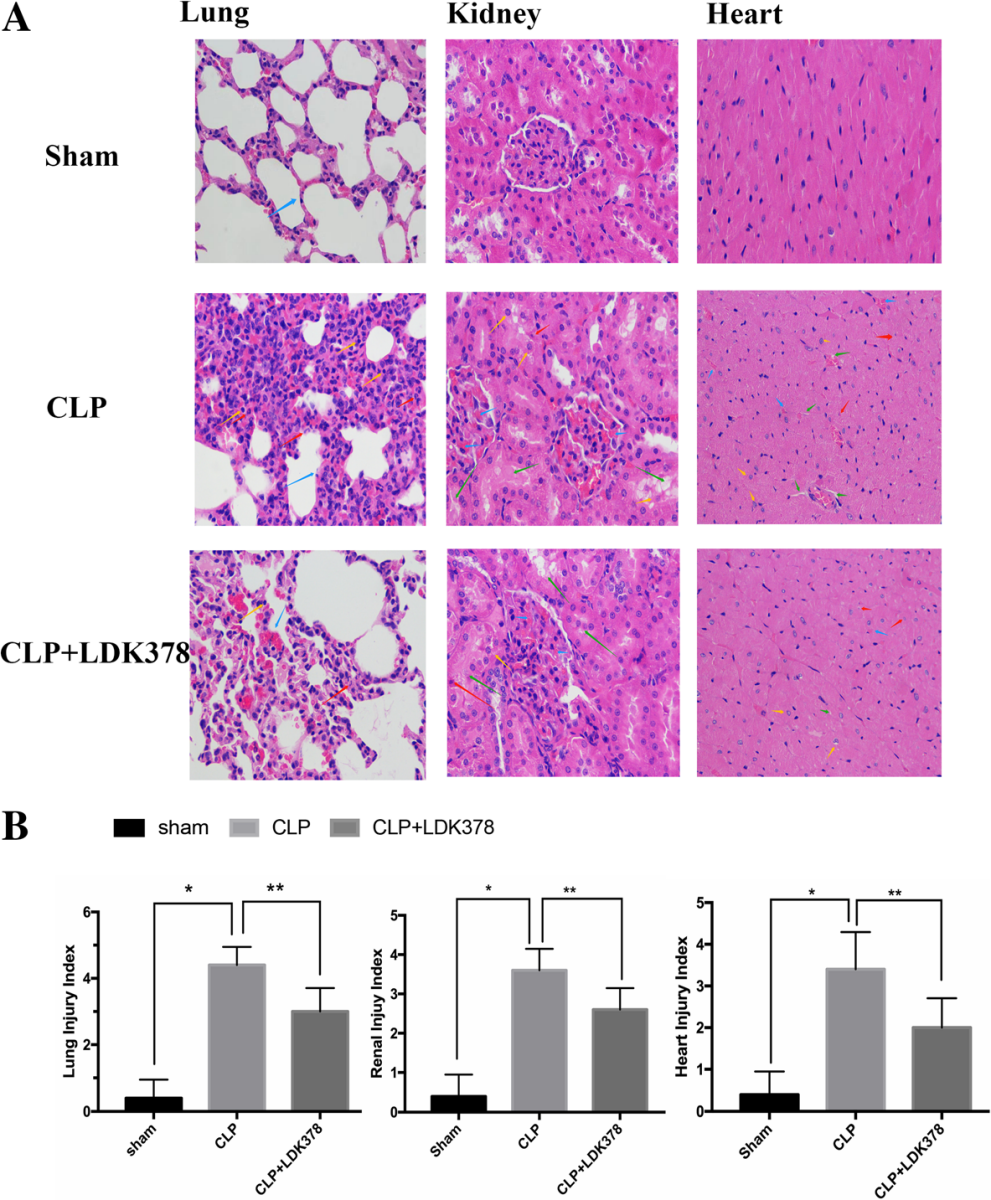
Morphological changes in major organ tissue pathology (400X). (**a**) Representative hematoxylin and eosin staining results for lung and kidney sections in rats. (**b**) Semi-quantitative analysis of H&E staining. *p < 0.05 vs sham group; #*p* < 0.05 vs CLP group. Lung: Inflammatory cells were diffusely infiltrated, and the alveolar wall was significantly widened in CLP group; a smaller number of inflammatory cells infiltrated in the LDK378 treatment group, and the alveolar wall was slightly widened, individual inflammatory cells infiltrated in Sham group, and no obvious broadening of the alveolar wall was observed (red arrow: neutrophils; yellow arrow: lymphocytes; blue arrow: alveolar wall). Kidney: Hyaline degeneration of renal tubular epithelial cell, necrosis, shedding and hemorrhage in CLP group; Lesions were also seen in the LDK378 treatment group, but range was smaller than CLP group. (yellow arrow: hyaline degeneration: red arrow, necrosis; green arrow: shedding; blue arrow: hemorrhage). Heart: Increased cell gap, necrosis, hemorrhage was seen in CLP group; the number of necrosis and hemorrhage were smaller in LDK378 treatment group (green arrow: cell gap increased; yellow arrow: Cell degeneration; red arrow: necrosis; blue arrow: hemorrhage)

Hematoxylin and eosin staining revealed severe lung and kidney injuries (Fig. 3) in the CLP group, but the pathological changes in the LDK378 injection group were improved by comparison. Semi-quantitative assessment of the histological lesions showed a significantly higher score in the CLP group than in the sham and CLP + LDK378 groups (*p* < 0.05).

### Survival curves

**Fig. 4:**
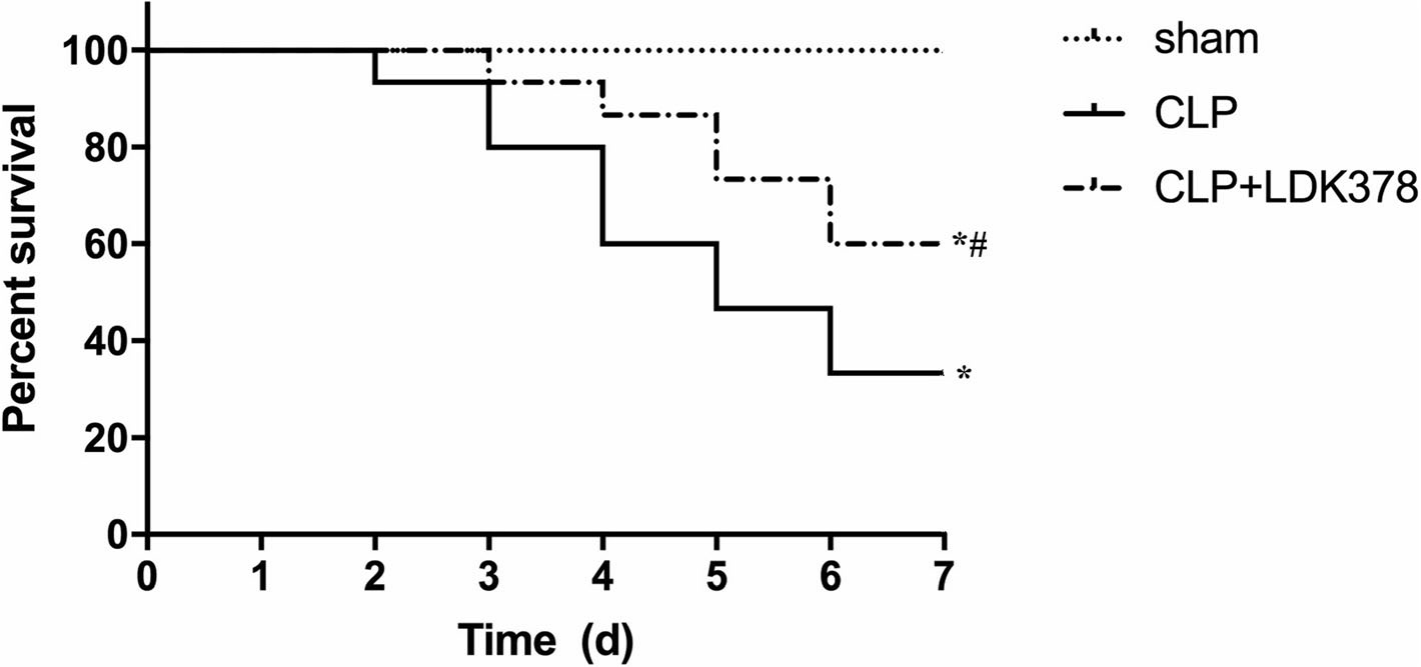
Survival outcomes among sham, CLP, and CLP + LDK378 groups. *p < 0.05 compared with sham group; #*p* < 0.05 compared with CLP-induced sepsis group

All of animals in the sham groups survived the entire 7-day experimental period. In CLP group, overall survival rate was 33.3% (5 out of 15). Survival in LDK378 injection group was 60% (9 out of 15). All the rats survived in the Sham group. The survival rate in the LDK378 treatment group was markedly greater than in the CLP group (*p* < 0.05) (Fig. 4).

## Discussion

The balance of inflammatory mediators is closely related to the severity and outcome of sepsis [19, 20]. Inflammatory cytokines play an important role in sepsis, as interplay between the initial inflammatory, and later, the anti-inflammatory responses lead to sepsis-induced organ dysfunction and lethality. Release of pro-inflammatory cytokines, particularly TNF-a, IL-6 is an important component of the host immune response and the role of these molecules in the pathogenesis of sepsis has been well studied. Overproduction of pro-inflammatory cytokines has been demonstrated and concentrations shown to correlate with severity and outcome of sepsis. In sepsis, inflammatory stimuli also activate counter-inflammatory cytokines such as IL-10 that can downregulate the host inflammatory response, and may have a key role to play in controlling the pro-inflammatory cytokine response. In the present study, CLP-induced sepsis caused an inflammatory response and damage in the heart, lung and kidney. The treatment with LDK378 injection ameliorated the pathological changes in main organic tissues caused by sepsis. Furthermore, we demonstrated the inhibitory effects of LDK378 on the sepsis-induced up-expression of TNF-a and IL-6 and the enhanced effects of LDK378 on IL-10 expression in septic rats. These data suggested that the protective effect of an LDK378 injection on organic injury might occur via the regulation of the secretion of inflammatory cytokines.

Because a common event in STING activation by different ligands is the phosphorylation of TBK1, the carboxy1 terminus of STING containing just 39 amino acids is necessary and sufficient to activate TBK1. Furthermore, STING not only activates TBK1 but also recruits IRF3 to TBK1 to activate the IRF3 pathway. Therefore, we examined STING by the expression and phosphorylation of TBK1. STING promotes the activation of TANK binding kinase 1 (TBK1), leading to IFN regulatory factor 3 (IRF-3) translocating into the nucleus for type I IFN transcription. In addition, the activation of STING induces its dimerization and ubiquitination, which are proposed to play important roles in the activation of IRF-3 signaling [21]. The activation of STING facilitates the recruitment of IRF-3 and TBK-1 into a complex where IRF-3 is phosphorylated. Phosphorylated IRF-3 forms dimers and is transported to the nucleus to activate transcription of type I IFN genes [21].

Anaplastic lymphoma kinase (ALK), a tumor-associated receptor tyrosine kinase, is an enzyme which in humans is encoded by the ALK gene. ALK is a member of the insulin receptor superfamily and shows the greatest sequence similarity to leukocyte tyrosine kinase (LTK), which is a receptor protein-tyrosine kinase. Twenty different ALK-fusion proteins that result from various chromosomal rearrangements have been identified, and they have been implicated in the pathogenesis of several diseases including anaplastic large-cell lymphoma, diffuse large B-cell lymphoma, and inflammatory myofibroblastic tumors [22]. A previous study found that both STING-KO mice and TRIF-KO mice were protected from sepsis in a severe CLP model [15], and the ALK-STING pathway was upregulated more in a human sepsis group than a control group. Both pharmacological and genetic disruption of ALK expression can diminish the stimulator of interferon gene-mediated host immune responses to CDNs in monocytes and macrophages [16]. These findings uncover that ALK plays a key role in modulating the inflammatory signaling pathway. But there is no direct interaction between ALK and phosphorylation of core components of the STING pathway. Zeng [16] found that the phosphorylation of epidermal growth factor receptor (EGFR) was up-regulated by 3′3’-cGAMP and c-di-AMP and ALK-EGFR-AKT pathway is a critical driver of STING activation in innate immune cells, the interplay between ALK and EGFR contributes to the AKT-dependent STING activation in macrophages and monocytes. In the present study, LDK378 treatment reduced p-TBK1 and its downstream transcription factors including phosphorylation of IRF3 and NF-kB in CLP-induced septic rats [23, 24]. Transcription factors induce the production of pro-inflammatory cytokines and type I interferon, indicating that LDK378 can regulate the secretion of inflammatory cytokines through its upstream transcription factors.

Microcirculation is comprised of arterioles, capillaries, and draining venules, and it performs vital functions including oxygen delivery and solute exchange. The inflammatory responses to an infection of microcirculation alter regional blood flow, vascular hyperpermeability, leukocyte recruitment, and coagulation [25]. Microcirculation stasis in low-flow states is enhanced by cytokine-stimulated monocytes and endothelial cells, and diminished deformability of red and white blood cells. Inflammation, ischemia, and reperfusion activate endothelial cells, leukocytes, and platelets, which produce oxygen radicals and inflammatory mediators, causing increased vascular permeability. Thus, a self-enhancing reduction in microcirculatory blood flow occurs, which leads to organ failure. The severity of impaired microcirculation is closely related to the severity of sepsis. Hua, Tianfeng [4] found a close relationship between proinflammatory cytokines and microcirculatory parameters and proved that proinflammatory cytokines generated an intense response that impairs microcirculation. In the present study, LDK378 treatment was shown to improve microcirculation, in which a possible mechanism might be by reducing the cytokine release of CLP-induced sepsis, hence, LDK378 may play a vital role in attenuating the organic injury or the fatality rate.

This study has some limitations. First, we only used moderate septic models in the study; severe models characterized by sepsis onset and rapid progression to multi-organ failure were not examined. Second, the study did not investigate the effects of different concentrations of LDK378 on sepsis. The optimal dose and time point of LDK378 administration were not well established. Therefore, further studies are needed to fully develop LDK378 as a therapeutic strategy for the treatment of sepsis. Third, the study did not discuss in-depth the mechanism of the ALK-STING pathway.

## Conclusions

LDK378 alleviates dysfunctions in micro- and macro-circulation of septic rats, thereby mitigating organic injuries and improving the survival rate. The protective effect of LDK378 reduces inflammatory injuries by regulating the ALK-STING pathway. This study provides valuable insights into the role of LDK378 in controlling inflammatory responses and reveals its therapeutic potential for inflammatory disorders.

## Methods

### Ethical statement

Sixty healthy male Sprague-Dawley (SD) rats weighing 400–450 g were obtained from the Experimental Animal Center of Traditional Chinese Medicine, University of Guangzhou. All animals were cared for humanely and in compliance with the “Principles of Laboratory Animal Care” formulated by the National Society for Medical Research and the Guidelines. For the Care and Use of Laboratory Animals, prepared by the Institute of Laboratory Animal Resources and published by the National Institutes of Health (8th edition; Washington DC, National Academic Press, 2011). The protocol was approved by the Institutional Animal Care and Use Committee of the Tang Wanchun Laboratories of Emergency Critical Care Medicine, Sun Yat-sen Memorial Hospital, Sun Yat-sen University.

### Animal preparation

After inhalation of CO_2_ for 10 s, the animals were anesthetized by an intraperitoneal injection of pentobarbital (45 mg/kg) and additional doses (10 mg/kg) were administrated at intervals of approximately 1 h, or when required, to maintain anesthesia. After anesthesia, the lower quadrants of the abdomen were shaved and the surgical area was disinfected. The trachea was orally intubated with a 14-G cannula mounted on a blunt needle (Abbocath-T; Abbott Hospital Products Division, North Chicago, IL, USA) with a 145-degree angled tip. The animals breathed spontaneously. End-tidal CO_2_ was continuously monitored with a side-stream infrared CO_2_ analyzer (Model 200; Instrument Laboratory, Lexington, MA, USA). A PE-50 catheter (Becton Dickinson, Franklin Lakes, NJ, USA) was advanced into the descending aorta from the femoral artery for measurement of arterial pressure and sampling of arterial blood for detection of serum cytokines.

All the catheters were flushed intermittently with saline containing 2.5 IU/ml of crystalline bovine heparin. A small midline abdominal incision was made, and after intramuscular, fascial, and peritoneal incisions, the cecum was located and exteriorized. Total cecal length was measured from the tip of the ascending cecum to the tip of the descending cecum. The cecum was immediately ligated with 4–0 silk at 50% of its total length, and distal to the ileocecal valve, without causing intestinal obstruction. The cecum was then punctured twice with a 20-gauge needle. After removing the needle, a small amount of feces were extruded. The cecum was relocated, and the abdomen was closed in two layers. Trained professionals monitored the rats 3 times a day after the surgery until the end of the experiment. For the survival study, animals were constantly monitored for 7 days. The survival rate was recorded every 24 h.

### Experimental protocol

A total of 60 rats were randomized into three groups: a sham group, CLP group and CLP + LDK378 group (*n* = 20 in each group). Five rats were randomly selected from each group for the mechanism study; the remaining 15 rats in each group were involved in a survival curve examination. LDK378 (20 mg/kg) was dissolved in a vehicle [10% dimethyl sulfoxide, 20% cremophor/ethanol (3:1), and 70% phosphate-buffered saline (PBS)] [16] and repeatedly administered via intraperitoneal injection to rats at 0 h, 24 h, and 48 h after CLP [26]. The sham group rats were anesthetized and underwent laparotomy without CLP. Rats in the sham and CLP groups received the vehicle intraperitoneally without LDK378. Blood biochemistry and sublingual microcirculation, including perfused small vessel density (PVD) and microcirculatory flow index (MFI), were examined at 6 h, 30 h, and 54 h. The animals were euthanized at 54 h, and heart, lung, and kidney tissues were harvested for western blot and histological examination analyses. Histopathological changes in the heart, lungs, and kidneys were compared between subgroups. Additionally, the 7-day survival outcome was continuously monitored at 24-h intervals.

### Mean arterial pressure measurement

Mean arterial pressure, core temperature, and end-tidal (ETCO_2_) values were continuously recorded on a personal computer-based data acquisition system supported by WINDAQ software (DATAQ instruments, Akron, Ohio).

### Microcirculation examination

Microcirculation images of the sublingual area were obtained using a side stream dark-field imaging device (MicroScan; MicroVision Medical Inc. Amsterdam, The Netherlands) at 6 h, 30 h, and 54 h. A semiquantitative method was used to classify the blood flow of each small vessel [27, 28] as follows: 0 = no flow or microthrombosis; 1 = intermittent flow (absence of flow at least 50% of the time); 2 = sluggish flow; and 3 = continuous flow. Small vessels with blood flow scores of 2 or 3 were considered perfused small vessels. For the microcirculatory flow index (MFI) score calculation, the image was divided into four quadrants, and the predominant type of flow was assessed in small vessels (less than 20um in diameter) in each quadrant. The MFI score represents the average values of the four quadrants. Perfused vessel density (PVD) was calculated as the number of small perfused vessels crossing the lines, divided by the total length of the lines. The vessel size was measured with a micrometer scale superimposed on the video display. Two independent observers analyzed all recordings.

### Detection of serum cytokines

Homogenized blood samples from the different groups, sampled at 6 h, 30 h, and 54 h after CLP surgery, were centrifuged at 3000 x g for 10 min at 4 °C to obtain a supernatant. The levels of TNF-α, IL-6, and IL-10 in the blood samples were analyzed using commercial ELISA kits specific for rats (R&D Systems, Minneapolis, MN), according to the manufacturer’s protocol. Cytokine levels were expressed in units of pg/ml.

### Western blot

Heart, lung and kidney tissues from different groups, sampled at 54 h, were homogenized in lysis buffer (P0013B, Beyotime, Shanghai, China) centrifuged at 13,000 x g for 10 min at 4 °C to generate a supernatant containing the extracted protein. The protein concentration was measured using a bicinchoninic acid (BCA) protein assay kit (P0009, Beyotime, Shanghai, China). A 50 μg portion of each sample was electrophoresed on polyacrylamide gel (10%) and transferred onto a polyvinylidene difluoride membrane (EMD Millipore, Bedford, MA, USA). After blocking with blocking buffer, the blots were incubated over night at 4 °C with diluted primary antibodies against IRF3 (CST, Shanghai, China, 1:1000), phosphorylated IRF3 (CST, Shanghai, China, 1;1000), TBK1 (CST, Shanghai, China, 1;1000), phosphorylated TBK1 (CST, Shanghai, China, 1;1000), NF-kB (p65) (CST, Shanghai, China, 1;1000), and phosphorylated NF-kB (p-p65) antibodies (CST, Shanghai, China, 1;1000). The membrane was then washed three times in tris-buffered saline with Tween-20 (0.1%) and incubated with HRP-conjugated anti-rabbit IgG antibody (dilution, 1:5000) for 1 h at room temperature. The membrane was again washed three times for 10 min each time, and finally the immunoreactive proteins were detected using an enhanced chemiluminescence western blotting detection kit. The GAPDH protein served as an internal control (CST, Shanghai, China, 1:6000).

### Histological examination analysis

Heart, lung, and kidney tissues were harvested at 54 h after the CLP surgery. The tissue samples were fixed in 10% formalin solution, embedded in paraffin and then sectioned. The tissue sections were stained with hematoxylin and eosin reagent and observed by using light microscopy. Myocardial injuries were characterized by atrophy of myocardial fibers, inflammatory cell infiltration, coagulative necrosis, and liquefactive necrosis: 0, no damage; 1 (mild), interstitial edema and localized necrosis; 2 (moderate), widespread myocardial cell swelling and necrosis; 3 (severe), necrosis with contraction bands and compressed capillaries, or 4 (highly severe), diffuse necrosis with contraction bands, compressed capillaries and hemorrhage. Lung injuries were characterized by diffuse reactions in alveolar walls, thickening of the alveolar, and infiltration of inflammatory cells (neutrophilic and mononuclear) in alveolar walls. The score is scaled from 0 to 5, representing the severity of lung injury as follows: 0 is the absence of injury; 1 represents ling injury; 2 represents moderate or intermediate injury; 3 represents widespread or extensive injury; 4 represents severe or intense injury. Kidney injuries included tubular cell necrosis, cytoplasmic vacuole, hemorrhage, and tubular dilatation, higher scores represent more severe damage in a four-score system: 0, histopathological changes < 10%; 1, =10–25%; 2,= 25–50%; 3, = 50–70%; and 4,=75–100%. All slides were reviewed blindly and scored using a semi quantitative scoring system [29–31].

### Survival curves

To evaluate the effect of LDK378 on survival outcome in septic rats, 45 rats were from the three groups: sham operation group (sham group, *n* = 15), CLP-induced sepsis group (CLP group, *n* = 15), and LDK378-treated sepsis group (CLP + LDK378group, *n* = 15), were continuously monitored for fatality at 24 h intervals for up to 7 days [28].

### Statistical analysis

Results were expressed as mean ± SD. Statistical analysis was performed using SPSS 25 software(SPSS Inc., Chicago, IL). Differences between groups were analyzed using one-way ANOVA tests. Survival was determined by Log-rank tests. A *p* value< 0.05 was considered statistically significant.

## Abbreviations

ALK: Anaplastic lymphoma kinase
CLP: Cecal ligation and puncture
ER: endoplasmic reticulum
ETCO2: end-tidal
IFN: type-I interferon
IRF3: Interferon regulatory factor 3
LTK: leukocyte tyrosine kinase
MFI: microcirculatory flow index
NSCLC: non-small cell lung cancer
PVD: perfused small vessel density
STING: Stimulator of interferon genes
TBK1: TANK binding kinase 1
